# Limb Ischemia in a Patient with Hyperosmolar Hyperglycemic State

**DOI:** 10.5811/cpcem.2018.9.39920

**Published:** 2018-10-17

**Authors:** Ahmed Al Hazmi, Sara Manning

**Affiliations:** * University of Maryland Medical Center, Department of Emergency Medicine, Baltimore, Maryland; † University of Maryland School of Medicine, Department of Emergency Medicine, Baltimore, Maryland

## Abstract

A 61-year-old male with a recent diagnosis of pemphigus vulgaris was brought to the emergency department for altered mental status. He had recently started taking prednisone to manage his autoimmune disease and had a progressive decline in his mental status along with decreased oral intake. Evaluation revealed hyperosmolar hyperglycemic state (HHS) and occlusive arterial thrombosis, a rare but known complication of HHS. He was resuscitated aggressively with intravenous fluids, insulin, and heparin and admitted to the intensive care unit. Emergency physicians should remain vigilant for ischemic complications in patients with HHS. Early recognition and treatment can reduce the morbidity and mortality associated with this endocrine emergency.

## INTRODUCTION

Hyperosmolar hyperglycemic state (HHS) is defined as severe hyperglycemia, a marked increase in serum osmolarity with clinical signs of severe dehydration in the absence of ketoacids.^1^ The hyperosmolar state is a serious hyperglycemic emergency that affects patients with type 2 diabetes mellitus and is associated with significant morbidity and mortality.^2^ About 1% of emergency department (ED) patients with diabetes-related conditions have HHS.^3^,^4^ The mortality rate associated with HHS is five times higher than that associated with diabetic ketoacidosis (DKA).^5^ The prognosis for patients with HHS is significantly worse at the extreme of ages and in the presence of hypoperfusion, especially coma.^6^

## CASE REPORT

A 61-year-old man was transported to the ED by ambulance with a complaint of confusion. His past medical history was significant for recently diagnosed pemphigus vulgaris that was being treated with steroids. His family described gradual functional decline over the prior two weeks with no changes in his skin lesion. On the day of ED presentation, he was no longer responding verbally to his family members. He had no history of diabetes mellitus or peripheral vascular disease.

On assessment in the ED, he was found to have a waxing and waning level of consciousness and was alert to self only. His initial vital signs were heart rate of 134 beats per minute, blood pressure of 141/86 millimeters of mercury, respiration of 16 breaths per minute, temperature of 37.1°C (98.8°F), and oxygen saturation of 95% on room air. He was ill-appearing with dry mucous membranes and poor skin turgor. He was also found to have a cold, pulseless, right lower extremity. Laboratory evaluation revealed marked hyperclycemia (blood glucose, 1077 milligrams per deciliter [mg/dL]), hemoconcentration (hemoglobin, 20 grams/dL), sodium concentration of 172 millimoles/L, anion gap of 23, and acute kidney injury (creatinine, 2.47 mg/dL). Urinalysis revealed a urinary tract infection without ketones. His thyroid-stimulating hormone level and a noncontrast computed tomography of his head were unremarkable. Duplex ultrasound of the lower extremity demonstrated total occlusion of the right proximal common iliac and popliteal arteries.

Based on these results, we diagnosed HHS with limb ischemia and sepsis from urinary tract infection. The patient was treated with insulin, heparin, and broad-spectrum antibiotics in the ED. His free water deficit was calculated to be greater than 10 L. Fluid management included initial volume resuscitation with 3 L of Plasma-Lyte. He was then transitioned to slow sodium correction with normal saline over 24 hours. He was admitted to an intensive care unit (ICU) with vascular surgery consultation for management of the limb ischemia.

The patient underwent through-the-knee amputation due to irreversible tissue damage on hospital day three. During his ICU stay, his blood glucose and sodium levels were corrected gradually and the acute kidney injury resolved. The patient did well during hospitalization and was discharged to an inpatient rehabilitation facility on hospital day 11.

## DISCUSSION

The pathogenesis of HHS is not fully understood, but the syndrome is attributed to decreased insulin sensitivity coupled with increases in the counter-regulatory hormones—cortisol, catecholamines, growth hormone, and glucagon—all of which increase hepatic and renal glucose production.^6^ The hallmark of HHS is hyperglycemia leading to hyperosmolarity, which is secondary to dehydration from glucosuria and body fluid depletion caused by other precipitating factors. The level of hyperosmolarity best correlates with mental status changes in patients with HHS.^7^

In more than 50% of HHS cases, the precipitating factor is infection (e.g., pneumonia, urinary tract infection, or sepsis).^8^ Other precipitants are inadequate glucose control, surgery, cerebrovascular ischemia, and myocardial ischemia.^8^ Dehydration is more subtle, occurring slowly over the course of a few days. It is exacerbated by decreased oral intake, especially in the geriatric population.^9^ The use of corticosteroids has been linked to the development of hyperglycemia. Patients on high-dose steroids are particularly prone to HHS.^10^ The hyperosmolar state of HHS induces osmotic diuresis, leading to severe dehydration and fluid loss, which can be as high as 7–12 L, representing a 10–15% weight loss.^3^ The triad of hyperglycemia, hyperosmolar state, and dehydration leads to a catecholamine surge, with increased production of cortisol and glucagon, which worsens hyperglycemia and the hyperosmolar state.^1^

HHS develops over days to weeks. Its clinical manifestation starts as weakness and lethargy, progressing to obtundation and coma. These changes correlate with the plasma osmolality and usually become apparent at levels greater than 310 milliosmoles (mOsm) per kilogram.^11^,^12^ The physical examination usually reveals signs of dehydration (dry mucous membranes, dry axilla, decreased skin turgor, low jugular venous pressure, and hypotension^6^) with or without clinical evidence of the inciting event. The hyperosmolar state is a hypertonic state irrespective of the sodium level at presentation, because of intracellular sodium shifts. Measurable serum sodium levels can vary from hyponatremia to hypernatremia.^1^

At the onset of severe dehydration, intracellular fluid shift, and hyperglycemia, the serum sodium level does not reflect the body’s actual sodium level. The true sodium level is determined by adding 1.6 to the measured serum sodium value for every 100 mg/dL of glucose. If the uncorrected sodium level is normal or elevated, the patient has already lost a significant amount of fluid. The corrected sodium level reveals the true level of hypernatremia.^13^,^14^ Patients with HHS usually present with severe hyperglycemia (glucose >600 mg/dL). Those with end-stage renal disease can have levels exceeding 1000 mg/dL. These patients will also have hypokalemia and hypophosphatemia as a result of osmotic diuresis, leading to urinary loss of potassium and phosphate. These deficiencies are also associated with decreased oral intake.^14^

**CPC-EM Capsule**

What do we already know about this clinical entity?

Hyperosmolar hyperglycemic state (HHS) is a clinical syndrome characterized by a state of profound dehydration, metabolic derangements, and multiorgan system failure.

What makes this presentation of disease reportable?

This case highlights a prothrombotic state accompanying a state of profound dehydration in a patient with HHS.

What is the major learning point?

Thromboembolic events should be considered in patients with HHS.

How might this improve emergency medicine practice?

Early recognition of complications of HHS allows early involvement of specialized services, which can improve outcomes in this population.

The successful treatment of HHS has five cornerstones: volume correction, glycemic control, electrolyte management, addressing the underlying cause, and managing complications (Table). Fluid correction should be started immediately, as soon as the HHS diagnosis has been established. The goals of volume resuscitation are intravascular volume repletion, restoring normal plasma tonicity, and improving end-organ perfusion. The initial intravenous (IV) fluid of choice is 0.9% normal saline, administered with the goal of achieving 50% fluid resuscitation within the first 12 hours.^3^,^4^ After the initial resuscitation period, the patient’s clinical status should be reassessed so that the fluid choice can be adjusted based on plasma tonicity and electrolyte levels.^4^

Glycemic control is achieved with IV administration of insulin after fluid repletion. The recommended dose is 0.1 units per kilogram (U/kg). The goal is to decrease osmolarity to below 310 mOsm/kg and to achieve a glucose level less than 250 mg/dL. Normally, with IV fluids and tight glycemic control, HHS patients’ mental status improves rapidly. Once the blood glucose concentration is less than 250 mg/dL, the fluid can be switched to a dextrose-containing solution to avoid hypoglycemia while the patient is being bridged to subcutaneous insulin.^15^ Special attention should be paid to electrolytes as well. Typically, these patients present with hyponatremia secondary to hyperglycemic osmotic force that drives water into the vascular space, causing dilutional hyponatremia. In contrast, hypernatremia denotes profound dehydration. Volume repletion normally corrects the sodium disturbance.^9^,^16^

Additionally, the potassium level might be elevated despite a total body deficit secondary to potassium transit to the extracellular space, creating relative hyperkalemia without the acidosis typically seen with DKA. Before starting insulin therapy, it is imperative to reassess the potassium level and the need to start potassium replacement. Starting treatment with insulin may lead to hypokalemia due to the potassium shift back into the cell that accompanies volume resuscitation.^9^,^17^,^18^ Additionally, the total body phosphate level can be relatively low despite a normal or high phosphate level because of molecular physiology similar to that of hypokalemia. Phosphate should be replaced if the patient is found to be hypophosphatemic, as insulin will drive phosphate, along with potassium, into the cells. Given that phosphate is needed in all muscular contractions, its uncorrected low levels can lead to cardiac arrest.^9^,^19^,^20^

It is important to look for the factors that precipitated HHS and to correct them as quickly as possible. Ischemia of any organ can precipitate HHS. Myocardial ischemia, cerebrovascular accidents, bowel ischemia, and limb ischemia can cause catecholamine and cortisol surges that can worsen preexisting hyperglycemia. It is imperative to identify these factors and treat them.^7^–^10^

HHS can affect coagulation by increasing the levels of coagulation factors, especially protein C and factor VIII complex, and decreasing fibrinolytic activities.^21^ In a crossover study, Stegenga and associates showed that hyperglycemia itself promotes coagulation through activation of thrombin-antithrombin complexes. The same study demonstrated that hyperinsulinemia can increase plasminogen-activating factors that decrease fibrinolysis.^22^,^23^ Severe dehydration can induce vasoconstriction and thromboembolic events such as cerebrovascular accidents, myocardial infarction, arterial insufficiency in the lower extremities, and mesenteric ischemia.^24^–^26^

Severe dehydration and the prothrombotic state provoked by HHS can lead to end-organ ischemic events. This prothrombotic state can lead to acute myocardial ischemia, cerebrovascular accidents, acute mesenteric ischemia, and lower extremity ischemia. Patients who present with altered mental status and do not improve after initial resuscitative measures warrant further workup.^21^–^26^

Management of limb ischemia is aimed at saving limb as well as life, restoring blood flow, and preventing further cellular damage from thrombosis or embolism. While awaiting evaluation by the specialty service, patients should be started on anticoagulation with heparin. The current recommendation is a bolus of 80–150 U/kg followed by maintenance at 18 U/kg per hour. However, if heparin is contraindicated because of documented heparin-induced thrombocytopenia or antithrombin III deficiency, direct thrombin inhibitors can be given. The only exception to anticoagulation is if the patient is actively bleeding.^27^ It is imperative that emergent vascular surgical consultation be obtained for consideration of emergent vs. urgent revascularization if the limb is salvageable. In irreversible ischemia, amputation might be the only definitive treatment.^28^

## CONCLUSION

Emergency physicians should remain vigilant for ischemic complications in patients presenting with HHS. Early recognition and treatment can improve the morbidity and mortality rates associated with this endocrine emergency.

Documented patient informed consent and/or Institutional Review Board approval has been obtained and filed for publication of this case report.

## Figures and Tables

**Table:** Complications associated with HHS.^3^–^4^,^8^

Complications related to the disease process	Dehydration	Hyperviscosity Electrolyte derangement Metabolic acidosis Rhabdomyolysis Malignant hyperthermia
	Prothrombotic factor activation*	Cerebral infarcts Myocardial infarction Pulmonary embolism Acute respiratory distress syndrome Mesenteric vessel thrombosis Limb ischemia Disseminated intravascular coagulation
Complications related to therapy		Hypoglycemia Hypokalemia Cerebral edema Pulmonary edema Hyperchloremic non-anion gap acidosis

* Can be worsened by profound dehydration.
